# Fall prevention protocol for adult patients in an inpatient unit: implementation and maintenance costs*

**DOI:** 10.1590/1980-220X-REEUSP-2025-0138en

**Published:** 2025-09-29

**Authors:** Juliana Cristina da Silva Raimundo, Antônio Fernandes Costa Lima

**Affiliations:** 1 Universidade de São Paulo, Escola de Enfermagem, Programa de Pós-Graduação em Gerenciamento em Enfermagem, São Paulo, SP, Brazil.; 2 Universidade de São Paulo, Escola de Enfermagem, Departamento de Orientação Profissional, São Paulo, SP, Brazil.

**Keywords:** Hospital Units, Hospital Nursing Service, Accidental Falls, Security Measures, Direct Service Costs

## Abstract

**Objective:**

To analyze the average direct costs related to nursing professionals’ labor and materials required to implement and maintain a fall prevention protocol for adult patients in a Clinical and Surgical Inpatient Unit.

**Method:**

Quantitative, exploratory-descriptive research, of the single case study type, based on absorption microcosting, carried out from August to October/2024 in a Medium-sized General Private Hospital.

**Results:**

The direct cost for implementing the protocol totaled US$ 819.24, of which US$ 218.44 related to the direct labor of nursing professionals and US$ 600.80 to the acquisition of material resources. For protocol maintenance, the average total direct cost was US$ 1.77/patient for nursing activities and US$ 10.10 for direct monthly expenditure on materials.

**Conclusion:**

Knowledge of the financial aspects associated with nursing professionals and the acquisition of material resources aimed at implementing and maintaining an adult fall prevention protocol may support decision-making regarding the rational allocation of required resources.

## INTRODUCTION

According to the World Health Organization (WHO)^(1)^, 684,000 people die annually due to serious incidents caused by falls, making it the second leading cause of death from unintentional injury. Therefore, falls are considered a major public health problem, as people, regardless of age, are at risk, especially those with cognitive, sensory and physical mobility changes, mainly linked to aging associated with exposure to inadequate environments. The high costs associated with managing its repercussions stand out, for example, ranging from US$ 3,611 in the Republic of Finland to US$ 1,049 in Australia; in Canada, with the implementation of prevention measures, a reduction in the incidence of cases of up to 20% was observed, yielding a net savings of more than US$ 120 million/year^(1)^.

In a hospital setting, falls can result in brain hemorrhages, hip fractures, and in some cases, even death. They are considered preventable adverse events (AEs) with the adoption of simple preventive measures. To minimize its occurrence, WHO recommends prioritizing risk review and the application of prevention protocols^(2)^.

In 2021, WHO published a technical report broadly addressing the management and prevention of falls, called “Step safely – Strategies for preventing and managing falls across the life-course”. The report, which covers all age groups, when addressing the prevention of falls in older people during hospitalization, emphasizes measures to manage care risks, as people over 65 are the most affected by this AE^(3)^.

In Brazil, the National Patient Safety Program (*PNSP*)^(4)^ was created with the aim of promoting and supporting initiatives aimed at patient safety, involving patients and family members in the process; expanding access to safety information; producing and disseminating knowledge on the topic; and disseminating the topic in health education institutions. The PNSP also establishes the need to create and implement basic prevention protocols, considered risk management tools, and to define appropriate measures to promote healthcare safety and reduce the incidence of AEs, damages, and the tangible and intangible costs associated with them^(4)^.

However, the adoption of a given protocol requires investments in the adaptation of structural resources, the acquisition of materials, and the training of the professionals involved. Therefore, knowledge of the costs associated with these variables can support deliberative management bodies in relation to the decision-making process, aiming to increase the quality of care and the recommended safety requirements.

In this regard, considering the indispensability of nursing professionals, who provide uninterrupted assistance 24 hours a day in the hospital context, for the success of strategic actions to prevent the occurrence of AEs, this study is proposed with the aim of analyzing the average direct costs (ADC) related to the labor of nursing professionals and materials required for the implementation and maintenance of a protocol for preventing falls in adult patients in a Clinical and Surgical Inpatient Unit.

## METHOD

### Design of Study

This is a quantitative, exploratory/descriptive single case study.

### Local

The Medium-Sized General Private Hospital (*HPGMP*), the field of study, comprises an integrated network of 87 hospitals in the Supplementary Health System (*SSS*), distributed across several Brazilian states. Located in an inland city of the State of São Paulo, it serves users from this city, as well as those from other regions or states, which allows transfers between hospitals in the integrated network, for care with a higher level of complexity and specificity. Access to the services provided is at the user’s expense or through registration with a health plan from the network itself or from accredited providers. The HPGMP does not directly serve patients from the Brazilian Public Health System (*SUS*), except in cases of emergency and imminent risk of death.

The Clinical and Surgical Inpatient Unit (*UICC*) is intended for patients undergoing clinical and/or surgical treatment (elective or emergency); it has 24 inpatient beds, including ten rooms for accommodation in pairs of patients of the same biological sex (ward) and four individual apartments. It has a nursing assistant and four nursing technicians who work 44 hours a week, in 12-hour shifts, with one hour of rest.

The trainings *in situ* take place during the working day at the most appropriate time, established by the HPGMP Care Practice Nurse in agreement with the *UICC* Coordinating Nurse, with the frequency required to assimilate the content covered.

### Sample and Selection Criteria

Nursing professionals who participated in the training program for the implementation of the “Adult Fall Prevention Protocol” and who had at least one year of experience at *UIC*C were included in the study. Nursing professionals who were temporarily allocated to the *UICC* to cover time off or vacations were excluded. Since all nursing professionals at *UICC* met the selection criteria, the study population consisted of six Clinical Nurses (CN) and 26 Nursing Technicians (NT).

### Data Collection

In August/2024, meetings were held with the Care Practice Nurse and Coordinating Nurse to obtain information on *HPGMP* investments regarding direct labor (DL) of nursing professionals and acquisition of materials required in the process of implementing and maintaining the protocol at *UICC*. The aforementioned process consisted of two subprocesses: Nurses’ duties and NTs’ duties, both covering the activities established in the “Adult Fall Prevention Protocol”.

Considering the activities carried out by the CNs, in the subprocess, the Nurse’s duties were grouped, some of them by similarity, due to the short time spent on their execution. Thus, eight activities were established: “1) History taking and physical examination”; “2) Risk management and completion of the whiteboard”; “3) Daily guidance/Inspect bracelet/Install bracelet”; “4) Apply Morse scale”; “5) Develop the Nursing Care Systematization – *SAE*”; “6) Apply fall form”; “7) Prepare form/bracelet”; and “8) Mobilize patient/Assist with walking”. In the NT assignments subprocess, the previously mentioned grouping was also chosen. Therefore, three activities were obtained: “1) Daily guidance/Inspect bracelet/Install bracelet/Raise railings/Position bell”; “2) Mobilize patient/Assist with walking”; and “3) Make nursing notes”.

It is important to highlight that knowledge of the logical sequence of the activities that make up a given process helps in its understanding, contributing to its execution in the best possible way, and can even highlight items that need adjustments or reformulations^(5)^. In this sense, process mapping, still little researched in the health area, helps in better understanding complex systems and in adapting improvement interventions to their local context^(6)^.

From September to October/2024, non-participant observations were carried out of nursing assistants and nursing technicians during the performance of their respective activities provided for in the “Adult Fall Prevention Protocol”, in the morning, afternoon and evening periods, with the time spent (timed) on the actions under their responsibility and the consumption of materials being recorded.

The study was based on absorption microcosting, which provides for the identification of the real resources consumed by a patient or health service, allowing the determination of the real cost of providing health care by a given provider. Microcosting allows for the detailed definition of the variables that make up the costs, based on individual patient treatment data, with the review of medical records or clinical charts specifically for the study^(7)^. Absorption costing provides for the allocation of production costs to the goods produced; therefore, all production-related expenses are shared within the services or products performed^(8)^.

Thus, direct costs, defined as those that can be identified and quantified, which refer to a monetary value relative to the consumption of inputs (materials, medicines, solutions and DL)^(8)^, were calculated.

DL refers to professionals who work directly on a specific product or service, when it is possible to measure the time spent on its completion. It consists of salary, charges, vacation, and the year-end bonus^(9)^. In this study, DL was calculated based on salary information provided by the *HPGMP* Human Resources Department, complying with the anonymity and confidentiality of the professionals involved.

The direct cost of the variables in this study was calculated by multiplying the time spent by the executing professional by the cost of the respective DL, plus the cost of the materials used^(10)^.

To determine the average direct cost of the DL ${\bar{C(P_{t})}}_{mob}$ , the equation was used ${\bar{C(P_{t})}}_{mob}\;=\;\sum_{\;c=1}^{n}\left(\bar{t_{c}}\;\cdot \;\bar{Su_{c}}\right)$ , composed of the average time spent by each professional ( $\bar{t_{c}}$ ) and unit wage bill ( $\bar{Su_{c}}$ )^(10)^. Information regarding the costs of material acquisitions was provided by the Administrative Manager.

The values in reais (R$) were converted to US dollars (US$) at the average rate of US$ 5.46/R$ 1.00, based on the exchange rate of 08/31/2024, provided by the Brazilian Central Bank. Therefore, the average hourly and minute costs were US$ 5.32 and US$ 0.09 for the Clinical Nurse, US$ 5.19 and US$ 0.09 for the *UICC* Nurse Coordinator, US$ 5.10 and US$ 0.08 for the CN category, and US$ 2.75 and US$ 0.05 for the NT category.

### Data Analysis and Treatment

Data were organized in an electronic spreadsheet, using the software Microsoft Excel®, and then transported to the Direct Microcosting Simulator^(11)^, from the Research Group on the Economic Dimension of Nursing Management; and treated using descriptive statistics of position (mean, minimum, maximum) and scale (standard deviation – SD).

### Ethical Aspects

The Research Ethics Committee of the proposing Institution approved this study after the consent of the HPGMP Administration, through the consolidated opinion no. 6,928,764, dated 03/07/2024.

## RESULTS

For implementation of the “Adult Falls Prevention Protocol”, there were no structural modifications at the *UICC*, given that it had the necessary resources, such as adequate ambient lighting, a bedside bell, safety rails on the beds, grab bars, and non-slip flooring. At the time, only rolls of blue wristbands, whiteboards, and whiteboard markers were purchased, totaling US$ 600.80 (100.00%), with 96.20% corresponding to 20 whiteboards.

The Care Practice Nurse led the implementation of the “Adult Falls Prevention Protocol”. She received it from Head Office, took ownership of the content, and developed teaching resources to promote the nursing professionals training. Having graduated 21 years ago, she has a Specialization in Patient’s Quality of life and Safety and has worked at HPGMP for two years. She spent 18 hours on the implementation process, totaling US$ 95.76, of which US$ 21.28 (4 hours) related to the appropriation of the Protocol’s content, US$ 21.28 (4 hours) to the planning and preparation of the training aimed at UICC nursing professionals, and US$ 53.20 (10 hours) to the mediation/facilitation of 10 training sessions, each lasting 1 hour.

Of the 11 training sessions held, one was taught by the Coordinating Nurse (US$ 5.19), 26 NTs, eight CNs, and the Coordinating Nurse participated, totaling US$ 117.49; the highest cost corresponded to the DL of the NT category (US$ 71.50). In summary, Chart 1 shows that the total direct cost for implementing the Protocol totaled US$ 819.24 (100.00%), with 73.34% referring to the acquisition of material resources.

**Chart 1:** Distribution of total direct costs, in US dollars (US$*), covering the human and material resources required to implement the Adult Falls Prevention Protocol at UICC – Sao Paulo, SP, Brazil, 2024.

Resources	Total direct cost US$*	%
Care Practice Nurse and *UICC*Coordinator Nurse: training organization and delivery	100.95	12.32
Coordinating Nurse, Clinical Nurses and Nursing Technicians at *UICC*: participation in training	117.49	14.34
**Subtotal of direct human resources costs**	**218.44**	**26.66**
Materials for implementing the protocol	600.80	73.34
**TOTAL**	**819.24**	**100.00**

*Exchange rate: R$ 5.46/US$ 1.00. Central Bank, based on the quotation of 08/31/2024.

From September to October 2024, six Nurses and 26 NTs were observed during the activities recommended for maintaining the Adult Falls Prevention Protocol at UICC, in the morning, afternoon and evening periods – even and odd. All Nurses were female, with a mean age of 36.5 (SD = 3.86) years, a mean time since graduation of 8.16 (SD = 4.81) years, and a mean time working at *HPGMP* and *UICC* of 2.2 (SD = 3.09) years. The majority of NTs were female (84.62%), with a mean age of 39.4 (SD = 10.6) years, mean time since graduation of 10.2 (SD = 8.32) years, 5.64 (SD = 6.36) years of experience at *HPGMP*, and 5.52 (SD = 12.6) years of experience at *UICC*.

In the Nurse’s duties subprocess, each of the eight preventive activities was observed 75 times in the three shifts of the CNs’ work. The average time to complete the set of eight activities was 15.2 (SD = 2.3) minutes and the median was 14.9 minutes. The activities that took the longest average time were “5) Prepare the *SAE*” (5.5-SD = 2.1 minutes), “1) History taking and physical examination” (1.6-SD = 0.5 minutes) and “4) Apply the Morse scale” (1.6-SD = 0.4 minutes). The average times for activities “2) Risk management and completion of whiteboard”, “3) Daily orientation/Inspect wristband/Install wristband”, “6) Apply fall form” and “7) Prepare form/wristband” and “8) Mobilize patient/Assist with walking” were 1.1 (SD = 0.4), 1.2 (SD = 0.4), 1.4 (SD = 0.7), 1.4 (SD = 0.8) and 1.4 (SD = 0.1) minutes, respectively. The total cost was US$ 92.91, with an ADC of US$ 1.24 (SD = 0.19) and a median of US$ 1.21. Table 1 shows that the activities with higher ADC were “5) Prepare the *SAE*” (US$ 0.45 DP = 0.18), “1) History taking and physical examination” (US$ 0.13 DP = 0.03) and “4) Apply the Morse scale” (US$ 0.13 – DP = 0.03).

**Table 1:** Distribution of the costs, in US dollars (US$*), of the eight activities recommended for the maintenance of the Adult Falls Prevention Protocol at the UICC carried out by six CNs – São Paulo, SP, Brazil, 2024.

Activities (number of observations)	ADC	Standard deviation	Minimum cost	Maximum cost	Median
1) History taking and physical examination (n = 75)	0.13	0.04	0.08	0.24	0.12
2) Risk management and whiteboard completion (n = 75)	0.09	0.03	0.08	0.26	0.08
3) Daily Orientation/Inspect Bracelet/Install Bracelet (n = 75)	0.09	0.03	0.06	0.30	0.09
4) Apply Morse scale (n = 75)	0.13	0.03	0.07	0.22	0.12
5) Prepare the SAE (n = 75)	0.45	0.18	0.20	1.12	0.41
6) Apply fall form (n = 75)	0.11	0.06	0.06	0.29	0.09
7) Prepare form/bracelet (n = 75)	0.12	0.07	0.06	0.29	0.09
8) Mobilize patient/Assist with walking (n = 75)	0.11	0.01	0.10	0.12	0.11

*Exchange rate: R$ 5.46/US$ 1.00. Central Bank, based on the quotation of 08/31/2024.

In the NTs’ duties subprocess, each of the three preventive activities was observed 96 times in the three shifts of the NTs’ work. The average time to complete the set of three activities was 11.8 (SD = 3.80) minutes and the median was 11.10 minutes. The preventive activities taking the most time were: “1) Daily guidance/Inspect bracelet/Install bracelet/Raise railings/Position bell” (4.5–SD = 1.9 minutes) and “3) Make nursing notes” (4.4–SD = 1.9 minutes); the average time to complete the activity “2) Mobilize patient/Assist with walking” was 2.0 (SD = 1.9) minutes. The total cost was US$ 51.12, with an ADC of US$ 0.53 (SD = 0.17) and a median of US$ 0.50. According to Table 2, the activities with the highest ADC were “1) Daily orientation/Inspect bracelet/Install bracelet/Raise railings/Position bell” (US$ 0.20 SD = 0.09) and “3) Make nursing notes” (US$ 0.20 – SD = 0.07).

**Table 2:** Distribution of the costs, in US dollars (US$*), of the three activities recommended for the maintenance of the Adult Falls Prevention Protocol at the UICC carried out by 26 NTs – São Paulo, SP, Brazil, 2024.

Activities (number of observations)	ADC	Standard deviation	Minimum cost	Maximum cost	Median
1) Daily orientation/Inspect bracelet/Install bracelet/Raise railings/Position bell (n = 96)	0.20	0.09	0.09	0.54	0.18
2) Mobilize patient/Assist with walking (n = 96)	0.13	0.08	0.03	0.41	0.12
3) Make nursing notes (n = 96)	0.20	0.07	0.05	0.38	0.21

*Exchange rate: R$ 5.46/US$ 1.00. Central Bank, based on the quotation of 08/31/2024.

Considering the sets of preventive activities required to maintain the “Adult Falls Prevention Protocol” at UICC, carried out by CNs (ADC of US$ 1.24 – SD = 0.19) and NTs (ADC of US$ 0.53 – SD = 0.17), the total ADC with DL of nursing professionals corresponding to US$ 1.77 per patient was obtained. During all observations of the aforementioned sets of activities, the only material consumed was the blue bracelet (unit cost of US$ 0.01), totaling US$ 1.71 corresponding to the consumption of 171 units.

Finally, it should be clarified that the direct monthly cost of materials, established in the UICC quota, for the maintenance of the protocol was US$ 10.10, of which US$ 7.50 included 750 units of blue bracelets and US$ 2.60 included four units of whiteboard marker pens (unit cost of US$ 0.65).

## DISCUSSION

The preventive activities that are part of the subprocesses “Nurse’s duties” and “NT’s duties”, included in the implementation and maintenance process of the “Adult Fall Prevention Protocol” at *UICC*, were carried out by the CN and NT, as recommended; no aspects related to them that required any adjustments or reformulations were observed.

In healthcare, process mapping allows us to identify bottlenecks, errors, and opportunities for improvement, enabling more informed, data-driven decision-making. Therefore, it is an essential tool that contributes to the continuous improvement of the quality, safety and efficiency of the services provided^(6)^.

The growing adoption of continuous quality improvement initiatives in healthcare has generated an increase in interest in research to deepen its understanding. This is because such initiatives can have a significant impact on the quality of healthcare in several areas, focusing on improving the structure, the healthcare service delivery process, customer well-being, and reducing mortality^(12)^.

From this perspective, process-based management is essential to ensure quality care, optimize resources, and improve the experience for both patients and the teams of healthcare professionals involved. It proposes an approach for continuous improvement of processes to achieve the desired outcomes, contributing to the achievement of care and management results in hospital organizations, having a favorable impact on economic and financial aspects^(9)^.

To implement the “Adult Falls Prevention Protocol” at *UICC*, there were no structural changes, given that the Unit already had the necessary resources to adequately prevent falls. Through this study, the costs associated with the acquisition of materials and investment in planning and training of nursing professionals were initially demonstrated.

It is important to consider that promoting training, through the essential role of the Care Practice Nurse, who teaches specific training programs, has increased the provision of care by enabling the improvement of work activities, contributing to the continuous advancement of care quality and patient safety. The investment in training also allowed the allocation and rational use of resources, minimizing the occurrence of waste and resulting in the effectiveness and efficiency of the application of recommended fall prevention activities, which was observed during the non-participant observation periods.

Regarding the CN and NT DL required to maintain the Protocol, the financial investment of the *HGPM* was also highlighted. The relevance of carrying out preventive activities at the bedside is highlighted, whether in care management or in providing direct care to the patient. The relevance of risk management carried out daily by the CN stands out. Before the implementation of the Protocol, the risk of falling at *UICC* was signaled by means of blue spheres on the medical prescription and on the identification printed next to the patient’s bed, but without the application of any fall risk assessment scale.

After implementation, risk management became more accurate and individualized with the application of the Morse Falls Scale^(13)^. The proper application of this Scale is essential for managing the risk of falls, as it helps in the early identification of those most likely to fall, allowing the health team to adopt preventive measures early on by considering the history of falls, mental state and need for assistance, as well as enabling the personalization of the individualized approach, reducing the risk of incidents and promoting a safer environment^(14)^.

It is important to emphasize that the individualization of care, through *SAE*, directs and supports the work of nursing professionals, as the prescription of exclusive care for the prevention of falls in patients with medium and high risk has a favorable impact on the better use of DL by nursing professionals.

*SAE* consists of a valuable tool that helps define the role of the nurse, enabling them to use their technical-scientific and humanitarian knowledge in providing the care required by the individual, and to prove their professional practice with the operationalization of the Nursing Process, a method that directs the nurse’s clinical reasoning to guide the team they manage in relation to the care to be provided to the patient, their family, and specific groups^(15)^.

A systematic review and meta-analysis demonstrated that falls, despite being considered a preventable AE, continue to be a common problem in both public and private hospitals worldwide. It was found that, among the preventive interventions adopted (direct education of patients and/or professionals; modifications in the care environment; technological assistance devices; systems, service models, social context, leadership, policies or procedures to mitigate falls; rehabilitation, physiotherapy, physical activities or other therapeutic exercises; medication management; dietary modification), the education of patients and health professionals consisted of the most favorable strategy to prevent and reduce the occurrence of hospital falls^(16)^.

Therefore, the importance of teamwork and collaborative practice to achieve good results in preventing hospital falls is corroborated. However, for this to happen, health professionals need to acquire the skills required for collaborative action, with communication with patients/companions and interprofessional communication between members of the health team in the inpatient unit being highlighted^(17)^.

Given the high costs associated with falls among the elderly, from 2000 to 2020, a study carried out using the Hospital Information System of the Brazilian Public Health System underscored the need for investments in more effective measures to prevent and mitigate the damage caused by falls among the older people, favoring the prolongation of life with safety and quality, reducing the risk of falls, increasing public health initiatives with inclusive practices, such as the acquisition of furniture or equipment for daily use, resulting in a decrease in the number of hospitalizations^(18)^.

In the United States of America, a case-control study with 900,635 patients investigated the cost of a fall in a hospital environment and the cost-benefit of implementing a falls prevention program (*Tailoring Interventions for Patient Safety* – Fall TIPS), analyzing the period before, during and after its implementation. It was demonstrated that a fall without injury generated a burden of US$ 62,521.00 to the institution, of which US$ 35,365.00 were classified as direct costs; a fall with any type of injury corresponded to US$ 64,526.00. The total cost of implementing Fall TIPS was US$ 267,700.00 and contributed to the prevention of 567 falls, totaling savings of up to US$ 22,036.714^(19)^.

Finally, in addition to determining the costs related to the process of implementing and maintaining the “Adult Falls Prevention Protocol”, it is confirmed that prevention should always be prioritized by nurses^(20)^ and NTs, regardless of the associated costs, aiming to avoid the damage caused by the occurrence of this preventable AE, the resulting care consequences, and the intangible costs to the patient, which represent a change in the individual’s quality of life and the consequences of the disease itself, such as pain and suffering^(21)^.

### Implications for Practice

By highlighting the financial aspects associated with the nurses DL, in managerial and care positions, and NTs, as well as the acquisition of material resources required for the implementation and maintenance of an adult falls protocol in an Inpatient Unit, this micro-costing study contributes to the verticalization of knowledge on the subject. The method adopted can be reproduced in other hospital contexts and the results will support decision-making regarding the rational allocation of the required human and material resources.

### Study Limitations

Observations were made of fall prevention activities carried out only by CNs and NTs, in a single Inpatient Unit, which constitutes limitations of this study. For future studies, it is important to include other health professionals, who also develop fall prevention actions, and to increase the number of Units.

## CONCLUSION

The direct cost for implementing the “Adult Fall Prevention Protocol” at UICC totaled US$ 819.24, of which US$ 218.44 were related to the nursing professionals (Care Practice Nurse, Coordinating Nurse, CN, and NT) DL and US$ 600.80 were for the acquisition of materials (rolls of blue wristbands, whiteboards and whiteboard markers).

For the Protocol maintenance, the average total direct cost was US$ 1.77/patient for nursing activities and US$ 10.10 for direct monthly expenditure on materials. The preventive activities, performed by the CN, with higher ADC were “5) Prepare the *SAE*” (US$ 0.45/DP = 0.18), “1) History taking and physical examination” (US$ 0.13/DP = 0.03) and “4) Apply the Morse scale” (US$ 0.13/DP = 0.03). The preventive activities carried out by NTs, with the highest ADC, were: “1) Daily guidance/Inspect bracelet/Install bracelet/Raise railings/Position bell” (US$ 0.20 SD = 0.09) and “3) Make nursing notes” (US$ 0.20 SD = 0.07).

It is concluded that knowledge of the direct costs associated with the DL of nursing professionals and the acquisition/replacement of material resources may support decision-making regarding the rational allocation of resources required to successfully implement and maintain the “Adult Fall Prevention Protocol”.

## DATA AVAILABILITY

Not applicable. This is a single-case study whose data set supporting the results is not publicly available due to the presence of sensitive information and the need to preserve participant confidentiality. All relevant information for understanding the findings is described in the body of the article.
