# Supplementary material for: Fall prevention protocol for adult patients in an inpatient unit: implementation and maintenance costs
Source: Rev Esc Enferm USP. 2025 Sep 29;59:e20250138. doi: 10.1590/1980-220X-REEUSP-2025-0138en (PMC12478304; doi:10.1590/1980-220X-REEUSP-2025-0138en)
Supplement: Translation

# Translation: Protocolo de prevenção de quedas de pacientes adultos em unidade de internação: custos para implementação e manutenção*

**DOI:** 10.1590/1980-220X-REEUSP-2025-0138pt

**Authors:** Juliana Cristina da Silva Raimundo, Antônio Fernandes Costa Lima

**Affiliations:** 1 Universidade de São Paulo, Escola de Enfermagem, Programa de Pós-Graduação em Gerenciamento em Enfermagem, São Paulo, SP, Brasil.; 2 Universidade de São Paulo, Escola de Enfermagem, Departamento de Orientação Profissional, São Paulo, SP, Brasil.

**Keywords:** Unidades Hospitalares, Serviço Hospitalar de Enfermagem, Acidentes por Quedas, Medidas de Segurança, Custos Diretos de Serviços

## Abstract

**Objetivo:**

Analisar os custos diretos médios relativos à mão de obra de profissionais de enfermagem e materiais requeridos à implementação e manutenção de um protocolo de prevenção de queda de pacientes adultos numa Unidade de Internação Clínica e Cirúrgica.

**Método:**

Pesquisa quantitativa, exploratório-descritiva, do tipo estudo de caso único, fundamentada no microcusteio por absorção, realizada de agosto a outubro/2024 em um Hospital Privado Geral de Médio Porte.

**Resultados:**

O custo direto para a implementação do protocolo totalizou US$ 819,24, sendo US$ 218.44 relativos à mão de obra direta dos profissionais de enfermagem e US$ 600.80 à aquisição de recursos materiais. Para a manutenção do protocolo, obteve-se o custo direto médio total de US$ 1.77/paciente referente às atividades de enfermagem e US$ 10.10 relativos ao gasto direto mensal com materiais.

**Conclusão:**

O conhecimento dos aspectos financeiros associados aos profissionais de enfermagem e à aquisição de recursos materiais visando à implementação e manutenção de um do protocolo de prevenção de queda do adulto poderá subsidiar a tomada de decisões relativas à alocação racional dos recursos requeridos.

## INTRODUÇÃO

De acordo com a Organização Mundial da Saúde (OMS)^(1)^, 684.000 pessoas morrem anualmente em virtude de graves ocorrências causadas por quedas, correspondendo à segunda causa de morte por lesão não intencional. Portanto, as quedas são consideradas um grande problema de saúde pública, pois as pessoas, independentemente da idade, estão sob este risco, em especial portadores de alterações cognitivas, sensoriais e de mobilidade física, principalmente atreladas ao envelhecimento associado à exposição a ambientes inadequados. Destacam-se os custos elevados associados ao manejo das suas repercussões, como exemplos, variaram de US$ 3611 na República da Finlândia a US$ 1049 na Austrália; no Canadá, com a implementação de medidas de prevenção, observou-se a diminuição da incidência de casos em até 20%, rendendo uma economia líquida de mais de US$ 120 milhões/ano^(1)^.

No ambiente hospitalar, a ocorrência de quedas pode resultar em hemorragias cerebrais, fraturas de quadril e, em alguns casos, até em morte. São consideradas eventos adversos (EA) evitáveis, com a adoção de medidas preventivas simples. Visando a minimização da sua ocorrência, a OMS recomenda a priorização da revisão dos riscos e a aplicação de protocolos de prevenção^(2)^.

Em 2021, a OMS publicou um relatório técnico abordando, amplamente, o gerenciamento e a prevenção de quedas, traduzido para o português como “Pise com segurança – Estratégias de prevenção e gestão da queda durante a vida”. O relatório, que engloba todas as faixas etárias, ao tratar da prevenção de queda em idosos durante o período de hospitalização enfatiza medidas de gerenciamento dos riscos assistenciais, pois pessoas acima de 65 anos são as mais afetadas por este EA^(3)^.

No Brasil, o Programa Nacional de Segurança do Paciente (PNSP)^(4)^ foi criado objetivando-se promover e apoiar iniciativas visando a segurança do paciente, envolvendo pacientes e familiares no processo; ampliar o acesso sobre as informações de segurança; produzir e difundir conhecimento sobre a temática; e disseminar o tema em instituições de ensino de saúde. O PNSP estabelece, ainda, a necessidade da criação e implantação de protocolos básicos de prevenção, considerados ferramentas de gerenciamento de risco, e da definição de medidas apropriadas para favorecer a segurança assistencial e diminuir a incidência de EAs, os danos e os custos tangíveis e intangíveis a eles associados^(4)^.

Contudo, a adoção de um determinado protocolo requer investimentos na adequação de recursos estruturais, na aquisição de materiais, e na capacitação dos profissionais envolvidos. Então, o conhecimento dos custos associados a essas variáveis pode subsidiar as instâncias gerenciais deliberativas com relação ao processo decisório, visando incrementar a qualidade assistencial e os requisitos de segurança preconizados.

Nessa direção, considerando a imprescindibilidade dos profissionais de enfermagem, que prestam assistência ininterruptamente nas 24 horas no contexto hospitalar, para o êxito de ações estratégicas preventivas da ocorrência de EAs, propõe-se este estudo objetivando analisar os custos diretos médios relativos à mão de obra de profissionais de enfermagem e materiais requeridos à implementação e manutenção de um protocolo de prevenção de queda de pacientes adultos numa Unidade de Internação Clínica e Cirúrgica.

## MÉTODO

### Tipo ou Desenho do Estudo

Pesquisa quantitativa, exploratório/descritiva, na modalidade de estudo de caso único.

### Local

O Hospital Privado Geral de Médio Porte (HPGMP), campo de estudo, compõe uma rede integrada de 87 hospitais do Sistema de Saúde Suplementar (SSS), distribuídos em vários estados brasileiros. Localizado em uma cidade do interior do Estado de São Paulo, atende usuários desta cidade, bem como os provenientes de outras regiões ou Estados, o que possibilita transferências, entre os hospitais da rede integrada, para atendimento com maior nível de complexidade e especificidade. O acesso aos serviços prestados ocorre mediante o custeio do usuário ou cadastro em plano de saúde da própria rede ou de credenciados. O HPGMP não atende diretamente pacientes provenientes do Sistema Único de Saúde (SUS), apenas em casos de emergência e risco iminente de morte.

A Unidade de Internação Clínica e Cirúrgica (UICC) destina-se a pacientes em tratamento clínico e/ou cirúrgico (eletivo ou de urgência); conta com 24 leitos de internação, sendo dez quartos para acomodações em duplas de pacientes do mesmo sexo biológico (enfermaria) e quatro apartamentos individuais. Conta com um enfermeiro assistencial e quatro técnicos de enfermagem que cumprem jornada de 44 horas semanais, em turnos de 12 por 36 horas, com uma hora de descanso.

Os treinamentos *in loco* ocorrem durante a jornada de trabalho no momento mais propício, estabelecido pela Enfermeira de Práticas Assistenciais do HPGMP em concordância com a Enfermeira Coordenadora da UICC, com a frequência requerida à assimilação do conteúdo abordado.

### Amostra e Critérios de Seleção

Foram incluídos no estudo os profissionais de enfermagem que participaram do programa de capacitação para a implementação do “Protocolo de Prevenção de Queda Adulto” e que possuíam, no mínimo, um ano de atuação na UICC. Excluíram-se os profissionais de enfermagem que estivessem alocados, temporariamente, na UICC, para a cobertura de folgas ou férias. Visto que todos os profissionais de enfermagem da UICC atendiam aos critérios de seleção, a população do estudo foi composta por seis Enfermeiras Assistenciais (EnfA) e 26 Técnicos de Enfermagem (TE).

### Coleta de Dados

Em agosto/2024, realizaram-se reuniões com a Enfermeira de Práticas Assistenciais e Enfermeira Coordenadora visando à obtenção de informações sobre os investimentos do HPGMP referentes à mão de obra direta (MOD) de profissionais de enfermagem e aquisição de materiais requeridos no processo de implementação e manutenção do protocolo na UICC. O referido processo foi composto por dois subprocessos: atribuições da Enfermeira e atribuições do TE, ambos abrangendo as atividades estabelecidas no “Protocolo de Prevenção de Queda Adulto”.

Considerando as atividades realizadas pelas EnfA, procedeu-se, no subprocesso, às atribuições da Enfermeira, o agrupamento de algumas delas, por similaridade, devido ao curto tempo despendido à sua execução. Assim, estabeleceram-se oito atividades: “1) Anamnese e exame físico”; “2) Gerenciamento de risco e preenchimento do quadro branco”; “3) Orientação diária/Inspecionar pulseira/Instalar pulseira”; “4) Aplicar escala de Morse”; “5) Elaborar a Sistematização da Assistência de Enfermagem – SAE”; “6) Aplicar termo de queda”; “7) Preparar termo/pulseira”; e “8) Mobilizar paciente/Auxiliar na deambulação”. No subprocesso atribuições do TE, também se optou pelo agrupamento, anteriormente mencionado. Então, obtiveram-se três atividades: “1) Orientação diária/Inspecionar pulseira/Instalar pulseira/Elevar grades/Posicionar campainha”; “2) Mobilizar paciente/Auxiliar na deambulação”; e “3) Realizar anotação de enfermagem”.

Importante destacar que o conhecimento da sequência lógica das atividades componentes de um determinado processo auxilia na sua compreensão, contribuindo para que seja executado da melhor maneira possível e pode, inclusive, evidenciar itens que precisem de ajustes ou reformulações^(5)^. Nessa direção, o mapeamento de processos, ainda pouco pesquisado na área da saúde, auxilia na melhor compreensão de sistemas complexos e na adaptação de intervenções de melhoria ao seu contexto local^(6)^.

De setembro a outubro/2024, realizaram-se observações não participantes dos enfermeiros assistenciais e técnicos de enfermagem nos momentos de realização das respectivas atividades previstas no “Protocolo de Prevenção de Queda Adulto”, nos períodos da manhã, tarde e noite, registrando-se o tempo despendido (cronometrado) nas ações de sua responsabilidade e o consumo de materiais.

O estudo fundamentou-se no microcusteio por absorção, o qual prevê a identificação dos recursos reais consumidos por um paciente ou serviço de saúde, possibilitando determinar o custo real de ofertar cuidados de saúde, por um determinado prestador. O microcusteio propicia definir, detalhadamente, as variáveis componentes dos custos, a partir de dados individuais do tratamento do paciente, com a revisão de prontuários ou de ficha clínica específica para o estudo^(7)^. O custeio por absorção prevê a apropriação dos custos de produção aos bens produzidos; portanto, todos os gastos referentes à produção são compartilhados entre os serviços ou produtos realizados^(8)^.

Assim, foram calculados os custos diretos, definidos como aqueles que podem ser identificados e quantificados, os quais se referem a um valor monetário relativo ao consumo de insumos (materiais, medicamentos, soluções e MOD)^(8)^.

A MOD diz respeito aos profissionais que trabalham diretamente sobre um determinado produto ou serviço, quando possível mensurar o tempo despendido à sua realização. É composta pelo salário, encargos, férias e 13º salário^(9)^. Neste estudo, calculou-se a MOD a partir das informações salariais fornecidas pelo Departamento de Pessoal do HPGMP, respeitando-se o anonimato e sigilo dos profissionais envolvidos.

Calculou-se o custo direto das variáveis deste estudo multiplicando-se o tempo despendido pelo profissional executante, pelo custo da respectiva MOD, acrescido do custo dos materiais utilizados^(10)^.

Para apuração do custo direto médio da MOD ${\bar{C(P_{t})}}_{mob}$ , utilizou-se a equação ${\bar{C(P_{t})}}_{mob}\;=\;\sum_{\;c=1}^{n}\left(\bar{t_{c}}\;\cdot \;\bar{Su_{c}}\right)$ , composta pelo tempo médio dispendido por cada profissional ( $\bar{t_{c}}$ ) e massa salarial unitária ( $\bar{Su_{c}}$ )^(10)^. As informações relativas aos custos das aquisições de materiais foram disponibilizadas pelo Gerente Administrativo.

Os valores em reais (R$) foram convertidos para o dólar americano (US$) pela taxa média de US$ 5,46/R$1,00, com base na cotação de 31/08/2024, fornecida pelo Banco Central do Brasil. Então, os custos médios hora e minuto foram de US$ 5.32 e US$ 0.09 para a Enfermeira de Práticas Assistenciais, de US$ 5.19 e US$ 0.09 para a Enfermeira Coordenadora da UICC, de US$ 5.10 e US$ 0.08 para a categoria EnfA, e de US$ 2.75 e US$ 0.05 para a categoria TE.

### Análise e Tratamento dos Dados

Os dados foram organizados em planilha eletrônica, utilizando-se o programa Microsoft Excel®, em seguida transportados para o Simulador de Microcusteio Direto^(11)^, do Grupo de Pesquisa Dimensão Econômica do Gerenciamento em Enfermagem; e tratados por intermédio de estatística descritiva de posição (média, mínimo, máximo) e de escala (desvio padrão – DP).

### Aspectos Éticos

O Comitê de Ética em Pesquisa da Instituição proponente aprovou, após a anuência da Administração do HPGMP, a realização deste estudo por meio do parecer consubstanciado nº 6.928.764, de 03/07/2024.

## RESULTADOS

Para implementação do “Protocolo de Prevenção de Quedas Adulto”, não houve modificações estruturais na UICC, tendo em vista que dispunha dos recursos necessários, como exemplos, iluminação ambiente adequada, campainha a beira leito, grades de segurança nos leitos, barras de apoio e piso antiderrapante. À época, foram adquiridos apenas rolos de pulseiras azuis, quadros brancos e canetas marcadoras de quadro branco, totalizando US$ 600,80 (100,00%), sendo 96,20% correspondentes a 20 quadros brancos.

A Enfermeira de Práticas Assistenciais protagonizou a implementação do “Protocolo de Prevenção de Quedas Adulto”, foi ela quem o recebeu da Matriz, se apropriou do conteúdo e desenvolveu os recursos didáticos visando favorecer a capacitação dos profissionais de enfermagem. Com 21 anos de formada, possui Especialização em Qualidade e Segurança do Paciente e atua no HPGMP há dois anos. Dispendeu 18 horas no processo de implementação, totalizando US$ 95.76, sendo US$ 21.28 (4 horas) relativos à apropriação do conteúdo do Protocolo, US$ 21.28 (4 horas) ao planejamento e elaboração do treinamento destinado aos profissionais de enfermagem da UICC e US$ 53.20 (10 horas) à mediação/facilitação de 10 treinamentos, com duração de 1 hora.

Dos 11 treinamentos realizados, um ministrado pela Enfermeira Coordenadora (US$ 5.19), participaram 26 TE, oito EA e a Enfermeira Coordenadora, totalizando US$ 117,49; o maior custo correspondeu à MOD da categoria TE (US$ 71.50). Em síntese, verifica-se, no Quadro 1, que o custo direto total para a implementação do Protocolo totalizou US$ 819.24 (100,00%), sendo 73,34% referentes à aquisição dos recursos materiais.

**Quadro 1:** Distribuição dos custos diretos totais, em dólares americanos (US$*), abrangendo os recursos humanos e materiais requisitados à implementação do Protocolo de Prevenção de Quedas Adulto na UICC – São Paulo, SP, Brasil, 2024.

Recursos	Custo direto total US$*	%
Enfermeira de Práticas Assistenciais e Enfermeira Coordenadora da UICC: organização e ministração de treinamentos	100.95	12.32
Enfermeira Coordenadora, Enfermeiras Assistenciais e Técnicos de Enfermagem da UICC: participação nos treinamentos	117.49	14.34
**Subtotal dos custos diretos com recursos humanos**	**218.44**	**26.66**
Materiais para implementação do protocolo	600.80	73.34
**TOTAL**	**819.24**	**100.00**

*Taxa de conversão: R$ 5,46/US$ 1.00. Banco Central, com base na cotação de 31/08/2024.

De setembro a outubro de 2024, foram observados seis EnfA e 26 TE durante a realização das atividades preconizadas para a manutenção do Protocolo de Prevenção de Quedas Adulto na UICC, nos períodos da manhã, tarde e noite – par e ímpar. Todas as EnfA eram do sexo feminino, com média de idade correspondente a 36,5 (DP = 3,86) anos, tempo médio de formação a 8,16 (DP = 4,81) anos, e tempo médio de atuação no HPGMP e na UICC de 2,2 (DP = 3,09) anos. A maioria dos TE era do sexo feminino (84,62%), com média de idade de 39,4 (DP = 10,6) anos, tempo médio de formação de 10,2 (DP = 8,32) anos, de atuação no HPGMP de 5,64 (DP = 6,36) anos e de atuação na UICC de 5,52 (DP = 12,6) anos.

No subprocesso atribuições da Enfermeira, cada uma das oito atividades preventivas foi observada por 75 vezes nos três turnos de atuação das EnfA. O tempo médio de realização do conjunto de oito atividades correspondeu a 15,2 (DP = 2,3) minutos e a mediana a 14,9 minutos. As atividades que despenderam maior tempo médio foram “5) Elaborar a SAE” (5,5–DP = 2,1 minutos), “1) Anamnese e exame físico” (1,6–DP = 0,5 minutos) e “4) Aplicar a escala de Morse” (1,6–DP = 0,4 minutos). Os tempos médios das atividades “2) Gerenciamento de risco e preenchimento do quadro branco”, “3) Orientação diária/Inspecionar pulseira/Instalar pulseira”, “6) Aplicar termo de queda” e “7) Preparar termo/pulseira” e “8) Mobilizar paciente/Auxiliar na deambulação” foram de 1,1 (DP = 0,4), 1,2 (DP = 0,4), 1,4 (DP = 0,7), 1,4 (DP = 0,8) e 1,4 (DP = 0,1) minutos, respectivamente. Obteve-se o custo total de US$ 92.91, com CDM de US$ 1.24 (DP = 0.19) e mediana de US$ 1.21. Consta da Tabela 1 que as atividades com maior CDM foram “5) Elaborar a SAE” (US$ 0.45 DP = 0.18), “1) Anamnese e exame físico” (US$ 0.13 – DP = 0.03) e “4) Aplicar a escala de Morse” (US$ 0.13 – DP = 0.03).

**Tabela 1:** Distribuição dos custos, em dólares americanos (US$*) das oito atividades preconizadas para a manutenção do Protocolo de Prevenção de Quedas Adulto na UICC realizadas por seis EnfA – São Paulo, SP, Brasil, 2024.

Atividades (número de observações)	CDM	Desvio padrão	Custo mínimo	Custo máximo	Mediana
1) Anamnese e exame físico (n = 75)	0.13	0.04	0.08	0.24	0.12
2) Gerenciamento de risco e preenchimento do quadro branco (n = 75)	0.09	0.03	0.08	0.26	0.08
3) Orientação diária/Inspecionar pulseira/Instalar pulseira (n = 75)	0.09	0.03	0.06	0.30	0.09
4) Aplicar escala de Morse (n = 75)	0.13	0.03	0.07	0.22	0.12
5) Elaborar a SAE (n = 75)	0.45	0.18	0.20	1.12	0.41
6) Aplicar termo de queda (n = 75)	0.11	0.06	0.06	0.29	0.09
7) Preparar termo/pulseira (n = 75)	0.12	0.07	0.06	0.29	0.09
8) Mobilizar paciente/Auxiliar na deambulação (n = 75)	0.11	0.01	0.10	0.12	0.11

*Taxa de conversão: R$ 5,46/US$ 1.00. Banco Central, com base na cotação de 31/08/2024.

No subprocesso atribuição do TE, cada uma das três atividades preventivas foi observada por 96 vezes nos três turnos de atuação dos TE. O tempo médio de realização do conjunto de três atividades foi de 11,8 (DP = 3,80) minutos e mediana de 11,10 minutos. As atividades preventivas que dispenderam maior tempo foram: “1) Orientação diária/Inspecionar pulseira/Instalar pulseira/Elevar grades/Posicionar campainha” (4,5–DP = 1,9 minutos) e “3) Realizar anotação de enfermagem” (4,4–DP = 1,9 minutos); o tempo médio de realização da atividade “2) Mobilizar paciente/Auxiliar na deambulação” foi de 2,0 (DP = 1,9) minutos. Obteve-se o custo total de US$ 51.12, com CDM de US$ 0.53 (DP = 0.17) e mediana de US$ 0.50. Conforme a Tabela 2, as atividades com maior CDM foram “1) Orientação diária/Inspecionar pulseira/Instalar pulseira/Elevar grades/Posicionar campainha” (US$ 0.20 – DP = 0.09) e “3) Realizar anotação de enfermagem” (US$ 0.20 – DP = 0.07).

**Tabela 2:** Distribuição dos custos, em dólares americanos (US$*), das três atividades preconizadas para a manutenção do Protocolo de Prevenção de Quedas Adulto na UICC realizadas por 26 TE – São Paulo, SP, Brasil, 2024.

Atividades (número de observações)	CDM	Desvio padrão	Custo mínimo	Custo máximo	Mediana
1) Orientação diária/Inspecionar pulseira/Instalar pulseira/Elevar grades/Posicionar campainha (n = 96)	0.20	0.09	0.09	0.54	0.18
2) Mobilizar paciente/Auxiliar na deambulação (n = 96)	0.13	0.08	0.03	0.41	0.12
3) Realizar anotação de enfermagem (n = 96)	0.20	0.07	0.05	0.38	0.21

*Taxa de conversão: R$ 5,46/US$ 1.00. Banco Central, com base na cotação de 31/08/2024.

Considerando os conjuntos de atividades preventivas requeridas à manutenção do “Protocolo de Prevenção de Quedas Adulto” na UICC, realizados por EnfA (CDM de US$ 1.24 – DP = 0.19) e TE (CDM de US$ 0.53 – DP = 0.17), obteve-se o CDM total com MOD dos profissionais de enfermagem correspondente a US$ 1.77 por paciente. Durante todas as observações dos conjuntos de atividades supracitados, o único material consumido foi a pulseira azul (custo unitário de US$ 0.01), totalizando US$ 1.71 correspondentes ao consumo de 171 unidades.

Por fim, esclarece-se que custo direto mensal com os materiais, estabelecidos em cota da UICC, para a manutenção do protocolo foi de US$ 10.10, sendo US$ 7.50 com 750 unidades de pulseira azul e US$ 2.60 com quatro unidades de caneta marcadora de quadro (custo unitário de US$ 0.65).

## DISCUSSÃO

As atividades preventivas integrantes dos subprocessos “atribuições da Enfermeira” e “atribuições do TE”, constantes do processo de implementação e manutenção do “Protocolo de Prevenção de Queda Adulto” na UICC, foram executadas pelas EnfA e TE, conforme o preconizado; não foram observados aspectos a elas referentes que demandassem quaisquer ajustes ou reformulações.

Na área da saúde, por intermédio do mapeamento de processos, é possível identificar gargalos, erros e oportunidades de melhoria, permitindo uma tomada de decisão mais informada e baseada em dados. Portanto, consiste em uma ferramenta essencial que contribui para a melhoria contínua da qualidade, segurança e eficiência dos serviços oferecidos^(6)^.

A crescente adoção de iniciativas de melhoria contínua da qualidade na saúde tem gerado um aumento no interesse em pesquisas para aprofundar sua compreensão. Isso porque tais iniciativas podem repercutir, significativamente, na qualidade da assistência à saúde em várias áreas, com foco na melhoria da estrutura, no processo de prestação de serviços de saúde, na melhoria do bem-estar do cliente e na redução da mortalidade^(12)^.

Nesta perspectiva, a gestão baseada em processos é fundamental para garantir um atendimento de qualidade, otimizar recursos e melhorar a experiência, tanto dos pacientes quanto das equipes de profissionais de saúde envolvidos. Propõe uma abordagem para melhoria contínua dos processos a fim de alcançar os desfechos desejados, contribuindo para o alcance dos resultados, assistenciais e gerenciais, em organizações hospitalares, repercutindo favoravelmente nos aspectos econômico-financeiros^(9)^.

Para implementação do “Protocolo de Prevenção de Quedas Adulto” na UICC, não houve modificações estruturais, tendo em vista que a Unidade já dispunha dos recursos necessários à adequada prevenção da ocorrência de quedas. Por meio deste estudo, demonstraram-se, inicialmente, os custos associados à aquisição de materiais e ao investimento no planejamento e viabilização da capacitação dos profissionais de enfermagem.

Importante considerar que favorecer a capacitação, por meio da atuação imprescindível da Enfermeira de Práticas Assistenciais, ministrante dos programas específicos de treinamento, incrementou a prestação de cuidados ao propiciar o aprimoramento do desempenho das atividades laborais, contribuindo para melhoria contínua da qualidade assistencial e segurança do paciente. O investimento na capacitação possibilitou, também, a alocação e o uso racional dos recursos, minimizando a ocorrência de desperdícios e resultando na efetividade e eficácia da aplicação das atividades preventivas de quedas preconizadas, o que foi constatado durante os momentos de observação não participante.

Com relação à MOD de EnfA e TE requeridos à manutenção do Protocolo, evidenciou-se, também, o investimento financeiro do HGPM. Ressalta-se a pertinência da realização das atividades preventivas à beira leito, seja no gerenciamento do cuidado ou na prestação de cuidado direto ao paciente. Destaca-se a relevância do gerenciamento de riscos realizado diariamente pela EnfA. Antes da implementação do Protocolo, o risco de queda na UICC era sinalizado por meio de esferas azuis na prescrição médica e na identificação impressa ao lado do leito do paciente, porém sem a aplicação de nenhuma escala de avaliação do risco de queda.

Após a implementação, o gerenciamento de risco tornou-se mais preciso e individualizado com a aplicação da Escala de Quedas de Morse^(13)^. A adequada aplicação desta Escala é fundamental para o gerenciamento de risco de quedas, pois auxilia na identificação precoce daqueles com maior probabilidade de cair, permitindo que a equipe de saúde adote, precocemente, medidas preventivas ao considerar o histórico de quedas, estado mental e necessidade de auxílio, bem como possibilita personalizar a abordagem individualizada reduzindo o risco de incidentes e promovendo um ambiente mais seguro^(14)^.

Ressalta-se que a individualização do cuidado, por meio da SAE, direciona e fundamenta a atuação dos profissionais de enfermagem, pois a prescrição de cuidados exclusivos para a prevenção de quedas em pacientes com médio e alto risco repercute favoravelmente na melhor utilização da MOD dos profissionais de enfermagem.

A SAE consiste em uma valiosa ferramenta que auxilia na definição do papel do enfermeiro, propiciando-lhe usar seus saberes técnico-científicos e humanitários na prestação de cuidados requeridos pelo indivíduo, e comprovar seu exercício profissional com a operacionalização do Processo de Enfermagem, método que direciona o raciocínio clínico do enfermeiro para conduzir a equipe que ele gerencia em relação ao cuidado a ser prestado para com o paciente, sua família e em grupos específicos^(15)^.

Revisão sistemática e metanálise demonstrou que as quedas, apesar de consideradas um EA evitável, continuam sendo um problema comum em hospitais, públicos e privados, em todo o mundo. Verificou que, dentre as intervenções preventivas adotadas (educação direta de pacientes e/ou profissionais; modificações no ambiente de cuidado; dispositivos tecnológicos de assistência; sistemas, modelos de serviço, contexto social, liderança, políticas ou procedimentos para mitigar quedas; reabilitação, fisioterapia, atividades físicas ou outros exercícios terapêuticos; gerenciamento de medicamentos; modificação da dieta), a educação de pacientes e de profissionais da saúde consistiu na estratégia mais favorável para evitar e reduzir a ocorrência de quedas hospitalares^(16)^.

Então, corrobora-se a importância do trabalho em equipe e da prática colaborativa para o alcance de bons resultados na prevenção da queda hospitalar, mas para isso os profissionais de saúde precisam apropriar-se das competências requeridas para o agir colaborativo, ressaltando-se a comunicação com os pacientes/acompanhantes e comunicação interprofissional entre os membros da equipe de saúde da unidade de internação^(17)^.

Frente aos custos elevados associados a quedas de idosos, no período de 2000 a 2020, estudo realizado a partir do Sistema de Informações Hospitalares do Sistema Único de Saúde brasileiro ressaltou a necessidade de investimentos em medidas mais efetivas para prevenir e mitigar os danos decorrentes das quedas em idosos, favorecendo o prolongamento da vida com segurança e qualidade, diminuindo os riscos de queda, aumentando as iniciativas da saúde pública com práticas inclusivas, como a aquisição de mobílias ou equipamentos de uso diário, resultando na diminuição do número de internações^(18)^.

Nos Estados Unidos da América, um estudo de caso-controle com 900.635 pacientes investigou o custo de uma queda em ambiente hospitalar e o custo-benefício da implantação de um programa de prevenção de quedas (*Tailoring Interventions for Patient Safety* Fall TIPS), sendo analisado o período antes, durante e após a sua implantação. Demonstrou-se que uma queda sem lesão gerou um ônus de US$ 62,521.00 à instituição, dos quais US$ 35,365.00 foram classificados como custos diretos; uma queda com qualquer tipo de lesão correspondeu a US$ 64,526.00. O custo total da implantação do Fall TIPS foi de US$ 267,700.00 e contribuiu para a prevenção de 567 quedas, totalizando uma economia de até US$ 22,036.714^(19)^.

Por fim, para além da apuração dos custos relativos ao processo de implementação e manutenção do “Protocolo de Prevenção de Quedas Adulto”, corrobora-se que a prevenção deve ser sempre priorizada pelos enfermeiros^(20)^ e TE, independentemente dos custos associados, visando evitar os danos causados pela ocorrência deste EA evitável, os desdobramentos assistenciais dele decorrentes e os custos intangíveis ao paciente, os quais representam a alteração na qualidade de vida do indivíduo e as consequências da doença em si, como a dor e o sofrimento^(21)^.

### Implicações Para a Prática

Ao evidenciar os aspectos financeiros associados à MOD de enfermeiros, em posições gerenciais e assistenciais, e TE, bem como à aquisição de recursos materiais requeridos para a implementação e manutenção de um protocolo de quedas adulto em Unidade de Internação, este estudo de microcusteio contribui com a verticalização do conhecimento sobre a temática. O método adotado poderá ser reproduzido em outros contextos hospitalares e os resultados subsidiarão a tomada de decisões relativas à alocação racional dos recursos humanos e materiais requeridos.

### Limitação do Estudo

Foram realizadas observações das atividades preventivas de quedas executadas apenas por EnfA e TE, em uma única Unidade de Internação, consistindo em limitações deste estudo. Para estudos futuros indica-se a relevância de abranger os demais profissionais de saúde que também desenvolvem ações preventivas de quedas e ampliar o número de Unidades.

## CONCLUSÃO

O custo direto para a implementação do “Protocolo de prevenção de queda Adulto” na UICC totalizou US$ 819.24, sendo US$ 218.44 relativos à MOD dos profissionais de enfermagem (enfermeira de Práticas Assistenciais, Enfermeira Coordenadora, EnfA e TE) e US$ 600.80 à aquisição de materiais (rolos de pulseiras azuis, quadros brancos e canetas marcadoras de quadro branco).

Para a manutenção do Protocolo, obteve-se o custo direto médio total de US$ 1.77/paciente referente às atividades de enfermagem e US$ 10.10 relativos ao gasto mensal com materiais. As atividades preventivas, realizadas por EnfA, com maior CDM, foram “5) Elaborar a SAE” (US$ 0.45/DP = 0.18), “1) Anamnese e exame físico” (US$ 0.13/DP = 0.03) e “4) Aplicar a escala de Morse” (US$ 0.13/DP = 0.03). As atividades preventivas executadas por TE, com maior CDM, foram: “1) Orientação diária/Inspecionar pulseira/Instalar pulseira/Elevar grades/Posicionar campainha” (US$ 0.20 – DP = 0.09) e “3) Realizar anotação de enfermagem” (US$ 0.20 DP = 0.07).

Conclui-se que o conhecimento dos custos diretos, associados à MOD dos profissionais de enfermagem e à aquisição/reposição de recursos materiais, poderá subsidiar as tomadas de decisão relativas à alocação racional dos recursos requeridos à viabilização exitosa do processo de implementação e manutenção do “Protocolo de prevenção de queda Adulto”.

## DISPONIBILIDADE DE DADOS

Não se aplica. Trata-se de um estudo de caso único cujo conjunto de dados que dá suporte aos resultados não está disponível publicamente devido à presença de informações sensíveis e à necessidade de preservação da confidencialidade dos participantes. Todas as informações relevantes para a compreensão dos achados estão descritas no corpo do artigo.

# Translation: Protocolo de prevención de caídas de pacientes adultos en unidades de hospitalización: costes de implementación y mantenimiento*

**DOI:** 10.1590/1980-220X-REEUSP-2025-0138es

**Authors:** Juliana Cristina da Silva Raimundo, Antônio Fernandes Costa Lima

**Affiliations:** 1 Universidade de São Paulo, Escola de Enfermagem, Programa de Pós-Graduação em Gerenciamento em Enfermagem, São Paulo, SP, Brasil.; 2 Universidade de São Paulo, Escola de Enfermagem, Departamento de Orientação Profissional, São Paulo, SP, Brasil.

**Keywords:** Unidades Hospitalarias, Servicio de Enfermería en Hospital, Accidentes por Caídas, Medidas de Seguridad, Costos Directos de Servicios

## Abstract

**Objetivo:**

Analizar los costos directos promedio relacionados con la mano de obra y los materiales de los profesionales de enfermería necesarios para implementar y mantener un protocolo de prevención de caídas para pacientes adultos en una Unidad de Hospitalización Clínica y Quirúrgica.

**Método:**

Investigación cuantitativa, exploratorio-descriptiva, del tipo estudio de caso único, basada en microcosteo de absorción, realizada de agosto a octubre/2024 en un Hospital General Privado de Tamaño Mediano.

**Resultados:**

El costo directo de la implementación del protocolo fue de US$819.24, de los cuales US$218.44 estuvieron relacionados con la mano de obra directa de los profesionales de enfermería y US$600.80 con la adquisición de recursos materiales. Para el mantenimiento del protocolo, el costo directo total promedio fue de US$1.77/paciente para actividades de enfermería y US$10.10 para el gasto directo mensual en materiales.

**Conclusión:**

El conocimiento de los aspectos financieros asociados a los profesionales de enfermería y la adquisición de recursos materiales destinados a implementar y mantener un protocolo de prevención de caídas en adultos pueden apoyar la toma de decisiones respecto a la asignación racional de los recursos requeridos.

## INTRODUCCIÓN

Según la Organización Mundial de la Salud (OMS)^(1)^, 684 000 personas mueren cada año a causa de accidentes graves provocados por caídas, lo que supone la segunda causa de muerte por lesiones no intencionales. Por lo tanto, las caídas se consideran un gran problema de salud pública, ya que las personas, independientemente de su edad, están expuestas a este riesgo, en especial aquellas con alteraciones cognitivas, sensoriales y de movilidad física, principalmente relacionadas con el envejecimiento asociado a la exposición a entornos inadecuados. Cabe destacar los elevados costos asociados al manejo de sus repercusiones, que, por ejemplo, variaron de 3611 dólares estadounidenses en la República de Finlandia a 1049 dólares en Australia; en Canadá, con la implementación de medidas de prevención, se observó una disminución de la incidencia de casos de hasta un 20%, lo que supuso un ahorro neto de más de 120 millones de dólares al año^(1)^.

En el entorno hospitalario, las caídas pueden provocar hemorragias cerebrales, fracturas de cadera y, en algunos casos, incluso la muerte. Se consideran eventos adversos (EA) evitables con la adopción de medidas preventivas sencillas. Con el fin de minimizar su ocurrencia, la OMS recomienda dar prioridad a la revisión de los riesgos y a la aplicación de protocolos de prevención^(2)^.

En 2021, la OMS publicó un informe técnico que aborda ampliamente la gestión y la prevención de las caídas, traducido al español como «Pise con seguridad: estrategias para la prevención y la gestión de las caídas a lo largo de la vida». El informe, que abarca todos los grupos de edad, al tratar la prevención de caídas en personas mayores durante el periodo de hospitalización, hace hincapié en las medidas de gestión de los riesgos asistenciales, ya que las personas mayores de 65 años son las más afectadas por este EA^(3)^.

En Brasil, el Programa Nacional de Seguridad del Paciente (PNSP)^(4)^ fue creado con el objetivo de promover y apoyar iniciativas destinadas a la seguridad del paciente, involucrando a los pacientes y sus familiares en el proceso; ampliar el acceso a la información sobre seguridad; producir y difundir conocimientos sobre el tema; y diseminar el tema en las instituciones de enseñanza de salud. El PNSP establece, además, la necesidad de crear e implementar protocolos básicos de prevención, considerados herramientas de gestión de riesgos, y de definir medidas adecuadas para favorecer la seguridad asistencial y reducir la incidencia de EA, los daños y los costos tangibles e intangibles asociados a ellos^(4)^.

Sin embargo, la adopción de un protocolo determinado requiere inversiones en la adecuación de recursos estructurales, en la adquisición de materiales y en la capacitación de los profesionales involucrados. Por lo tanto, el conocimiento de los costos asociados a estas variables puede subsidiar a las instancias gerenciales deliberativas en relación con el proceso de toma de decisiones, con el objetivo de incrementar la calidad asistencial y los requisitos de seguridad preconizados.

En este sentido, considerando la indispensabilidad de los profesionales de enfermería, que prestan asistencia ininterrumpida las 24 horas del día en el contexto hospitalario, para el éxito de las acciones estratégicas preventivas de la ocurrencia de EA, se propone este estudio con el objetivo de analizar los costos directos medios relacionados con la mano de obra de los profesionales de enfermería y los materiales necesarios para la implementación y el mantenimiento de un protocolo de prevención de caídas de pacientes adultos en una Unidad de Hospitalización Clínica y Quirúrgica.

## MÉTODO

### Tipo o Diseño del Estudio

Investigación cuantitativa, exploratoria/descriptiva, en la modalidad de estudio de caso único.

### Lugar

El Hospital Privado General de Medio Porte (HPGMP), campo de estudio, forma parte de una red integrada de 87 hospitales del Sistema de Salud Suplementaria (SSS), distribuidos en varios estados brasileños. Ubicado en una ciudad del interior del estado de São Paulo, atiende a usuarios de esta ciudad, así como a los procedentes de otras regiones o estados, lo que permite el traslado entre los hospitales de la red integrada para recibir atención con un mayor nivel de complejidad y especificidad. El acceso a los servicios prestados se realiza mediante el pago por parte del usuario o la inscripción en un plan de salud de la propia red o de centros acreditados. El HPGMP no atiende directamente a pacientes procedentes del Sistema Único de Salud (SUS), salvo en casos de emergencia y riesgo inminente de muerte.

La Unidad de Internación Clínica y de Cirugía (UICC) está destinada a pacientes en tratamiento clínico y/o quirúrgico (electivo o de urgencia); cuenta con 24 camas de hospitalización, siendo diez habitaciones para alojamiento en parejas de pacientes del mismo sexo biológico (enfermería) y cuatro apartamentos individuales. Cuenta con un enfermero asistente y cuatro técnicos de enfermería que trabajan 44 horas semanales, en turnos de 12 por 36 horas, con una hora de descanso.

La capacitación *in situ* se lleva a cabo durante la jornada laboral en el momento más propicio, establecido por la enfermera de prácticas asistenciales del HPGMP, de acuerdo con la enfermera coordinadora de la UICC, con la frecuencia necesaria para la asimilación del contenido abordado.

### Muestra y Criterios de Selección

Se incluyeron en el estudio los profesionales de enfermería que participaron en el programa de capacitación para la implementación del “Protocolo de Prevención de Caídas en Adultos” y que tenían, como mínimo, un año de experiencia en la UICC.

Se excluyó al personal de enfermería que estaba asignado temporalmente a la UICC para cubrir turnos o vacaciones. Dado que todo el personal de enfermería de la UICC cumplía los criterios de selección, la población del estudio estuvo compuesta por seis enfermeras asistenciales (EnfA) y 26 técnicos de enfermería (TE).

### Recolección de Datos

En agosto de 2024, se realizaron reuniones con la enfermera de prácticas asistenciales y la enfermera coordinadora con el fin de obtener información sobre las inversiones del HPGMP en mano de obra directa (MOD) de profesionales de enfermería y la adquisición de materiales necesarios para el proceso de implementación y mantenimiento del protocolo en la UICC. Dicho proceso se compuso de dos subprocesos: atribuciones de la enfermera y atribuciones del TE, ambos abarcando las actividades establecidas en el «Protocolo de Prevención de Caídas en Adultos».

Teniendo en cuenta las actividades realizadas por las EnfA, en el subproceso se procedió a las atribuciones de la enfermera, agrupando algunas de ellas por similitud, debido al poco tiempo que se dedicaba a su ejecución. Así, se establecieron ocho actividades: «1) Anamnesis y examen físico»; «2) Gestión de riesgos y cumplimentación de la pizarra blanca»; «3) Orientación diaria/Inspección de la pulsera/Colocación de la pulsera»; «4) Aplicación de la escala de Morse»; «5) Elaborar la Sistematización de la Asistencia de Enfermería (SAE)»; «6) Aplicar el término de caída»; «7) Preparar el término/la pulsera»; y «8) Movilizar al paciente/Ayudar en la deambulación». En el subproceso de atribuciones del TE, también se optó por la agrupación mencionada anteriormente. Así, se obtuvieron tres actividades: «1) Orientación diaria/Inspeccionar pulsera/Colocar pulsera/Levantar barandillas/Colocar timbre»; «2) Movilizar al paciente/Ayudar a caminar»; y «3) Realizar anotaciones de enfermería».

Es importante destacar que el conocimiento de la secuencia lógica de las actividades que componen un determinado proceso ayuda a su comprensión, contribuyendo a que se ejecute de la mejor manera posible e incluso puede poner de manifiesto elementos que necesitan ajustes o reformulaciones^(5)^. En este sentido, el mapeo de procesos, aún poco investigado en el área de la salud, ayuda a comprender mejor los sistemas complejos y a adaptar las intervenciones de mejora a su contexto local^(6)^.

De septiembre a octubre de 2024, se realizaron observaciones no participantes de los enfermeros y técnicos de enfermería en el momento de la realización de las respectivas actividades previstas en el «Protocolo de Prevención de Caídas en Adultos», por la mañana, por la tarde y por la noche, registrando el tiempo empleado (cronometrado) en las acciones de su responsabilidad y el consumo de materiales.

El estudio se basó en el microcosteo por absorción, que prevé la identificación de los recursos reales consumidos por un paciente o servicio de salud, lo que permite determinar el costo real de la prestación de cuidados de salud por parte de un proveedor determinado. El microcosteo permite definir, en detalle, las variables que componen los costes, a partir de datos individuales del tratamiento del paciente, con la revisión de los historiales o de la ficha clínica específica para el estudio^(7)^. El costeo por absorción prevé la asignación de los costes de producción a los bienes producidos; por lo tanto, todos los gastos relacionados con la producción se comparten entre los servicios o productos realizados^(8)^.

Así, se calcularon los costos directos, definidos como aquellos que pueden identificarse y cuantificarse, los cuales se refieren a un valor monetario relativo al consumo de insumos (materiales, medicamentos, soluciones y MOD)^(8)^.

La MOD se refiere a los profesionales que trabajan directamente en un determinado producto o servicio, cuando es posible medir el tiempo dedicado a su realización. Se compone del salario, las cargas sociales, las vacaciones y el decimotercer salario^(9)^. En este estudio, se calculó la MOD a partir de la información salarial proporcionada por el Departamento de Personal del HPGMP, respetando el anonimato y la confidencialidad de los profesionales involucrados.

Se calculó el costo directo de las variables de este estudio multiplicando el tiempo dedicado por el profesional ejecutante por el costo de la MOD correspondiente, más el costo de los materiales utilizados^(10)^.

Para determinar el costo directo medio de la MOD ( ${\bar{C(P_{t})}}_{mob}$ ), se utilizó la ecuación, ${\bar{C(P_{t})}}_{mob}\;=\;\sum_{\;c=1}^{n}\left(\bar{t_{c}}\;\cdot \;\bar{Su_{c}}\right)$ compuesta por el tiempo medio dedicado por cada profesional ( $\bar{t_{c}}$ ) y la masa salarial unitaria ( $\bar{Su_{c}}$ )^(10)^. La información relativa a los costos de adquisición de materiales fue facilitada por el Gerente Administrativo.

Los valores en reales (R$) se convirtieron a dólares estadounidenses (US$) al tipo de cambio medio de 5,46 US$/R$1,00, basado en la cotización del 31/08/2024, facilitada por el Banco Central de Brasil. Así, los costes medios por hora y minuto fueron de 5,32 US$ y 0,09 US$ para la enfermera de prácticas asistenciales, de 5,19 y 0,09 dólares para la enfermera coordinadora de la UICC, de 5,10 y 0,08 dólares para la categoría EnfA, y de 2,75 y 0,05 dólares para la categoría TE.

### Análisis y Tratamiento de los Datos

Los datos se organizaron en una hoja de cálculo electrónica, utilizando el programa Microsoft Excel®, y luego se transfirieron al Simulador de Microcosto Directo^(11)^, del Grupo de Investigación Dimensión Económica de la Gestión en Enfermería; y se trataron mediante estadísticas descriptivas de posición (media, mínima, máxima) y de escala (desviación estándar – DE).

### Aspectos Éticos

El Comité de Ética en Investigación de la institución proponente aprobó, tras el consentimiento de la Administración del HPGMP, la realización de este estudio mediante el dictamen fundamentado n.º 6.928.764, de 03/07/2024.

## RESULTADOS

Para la implementación del «Protocolo de Prevención de Caídas en Adultos», no se realizaron modificaciones estructurales en la UICC, ya que contaba con los recursos necesarios, como, por ejemplo, iluminación ambiental adecuada, timbre junto a la cama, barandillas de seguridad en las camas, barras de apoyo y piso antideslizante. En ese momento, solo se adquirieron rollos de pulseras azules, pizarras blancas y marcadores para pizarra blanca, por un total de 600,80 dólares (100,00%), de los cuales el 96,20% correspondió a 20 pizarras blancas.

La enfermera de prácticas asistenciales protagonizó la implementación del «Protocolo de prevención de caídas en adultos», fue ella quien lo recibió de la sede central, se apropió del contenido y desarrolló los recursos didácticos con el fin de favorecer la capacitación de los profesionales de enfermería. Con 21 años de licenciatura, cuenta con una especialización en Calidad y Seguridad del Paciente y trabaja en el HPGMP desde hace dos años. Dedicó 18 horas al proceso de implementación, por un total de 95,76 dólares, de los cuales 21,28 dólares (4 horas) corresponden a la apropiación del contenido del Protocolo, 21,28 dólares (4 horas) a la planificación y elaboración de la capacitación destinada a los profesionales de enfermería de la UICC y US$ 53.20 (10 horas) a la mediación/facilitación de 10 capacitaciones, con una duración de 1 hora.

De las 11 capacitaciones realizadas, una impartida por la enfermera coordinadora (5,19 dólares), participaron 26 TE, ocho EnfA y la enfermera coordinadora, por un total de 117,49 dólares; el mayor costo correspondió a la MOD de la categoría TE (71,50 dólares). En resumen, en la Cuadro 1 se observa que el costo directo total para la implementación del Protocolo ascendió a US$ 819,24 (100,00%), de los cuales el 73,34% correspondió a la adquisición de recursos materiales.

**Cuadro 1:** Distribución de los costos directos totales, en dólares estadounidenses (US$*), que abarcan los recursos humanos y materiales necesarios para la implementación del Protocolo de Prevención de Caídas en Adultos en la UICC. São Paulo – SP, Brasil, 2024.

Recursos	Coste directo total US$*	%
Enfermera de Práctica Asistencial y Enfermera Coordinadora de la UICC: organización e impartición de cursos de formación	100.95	12.32
Coordinadora de enfermería UICC, auxiliares de enfermería y técnicos de enfermería: participación en cursos de formación	117.49	14.34
**Subtotal costes directos de recursos humanos**	**218.44**	**26.66**
Materiales para la aplicación del protocolo	600.80	73.34
**TOTAL**	**819.24**	**100.00**

*Tasa de conversión: R$ 5,46/US$ 1,00. Banco Central, con base en la tasa de cambio del 31 de agosto de 2024.

De septiembre a octubre de 2024, se observaron seis EnfA y 26 TEs realizando las actividades recomendadas para el mantenimiento del Protocolo de Prevención de Caídas en Adultos en la UICC, en los periodos de mañana, tarde y noche – pares e impares. Todos los EnfA eran mujeres, con una edad media de 36,5 (DE = 3,86) años, un periodo medio de formación de 8,16 (DE = 4,81) años, y un tiempo medio de trabajo en el HPGMP y en la UICC de 2,2 (DE = 3,09) años. La mayoríade los TE eran mujeres (84,62%), con una edad media de 39,4 (DE = 10,6) años, un tiempo medio de formación de 10,2 (DE = 8,32) años, 5,64 (DE = 6,36) años trabajando en el HPGMP y 5,52 (DE = 12,6) años trabajando en la UICC.

En el subproceso Tareas de la enfermera, cada una de las ocho actividades preventivas se observó 75 veces en los tres turnos en los que trabajaban las enfermeras. El tiempo medio empleado en realizar las ocho actividades fue de 15,2 (DE = 2,3) minutos y la mediana de 14,9 minutos. Las actividades que llevaron más tiempo de media fueron “5) Elaboración del SNC” (5,5–DP = 2,1 minutos), “1) Anamnesis y exploración física” (1,6–DP = 0,5 minutos) y “4) Aplicación de la escala de Morse” (1,6–DP = 0,4 minutos). Los tiempos medios de las actividades “2) Gestión de riesgos y cumplimentación de la pizarra”, “3) Orientación diaria/Inspeccionar pulsera/Instalar pulsera”, “6) Aplicar término de caída” y “7) Preparar término/pulsera” y “8) Movilizar al paciente/Ayudar en la deambulación” fueron de 1,1 (DE = 0,4), 1,2 (DE = 0,4), 1,4 (DE = 0,7), 1,4 (DE = 0,8) y 1,4 (DE = 0,1) minutos, respectivamente. El coste total fue de 92,91 dólares, con una MDC de 1,24 dólares (SD = 0,19) y una mediana de 1,21 dólares. La Tabla 1 muestra que las actividades con mayor CDM fueron “5) Elaboración de la ESS” (0,45 US$ – DE = 0,18), “1) Anamnesis y exploración física” (0,13 US$ – DE = 0,03) y “4) Aplicación de la escala de Morse” (0,13 US$ – DE = 0,03).

**Tabla 1:** Distribución de los costos, en dólares estadounidenses (US$*) de las ocho actividades recomendadas para el mantenimiento del Protocolo de Prevención de Caídas en Adultos en la UICC realizadas por seis ENS – São Paulo,SP, Brasil, 2024.

Actividades (número de observaciones)	CDM	Desviación estándar	Coste mínimo	Máximo coste	Mediana
1) Anamnesis y exploración física (n = 75)	0.13	0.04	0.08	0.24	0.12
2) Gestión de riesgos y cumplimentación de la pizarra (n = 75)	0.09	0.03	0.08	0.26	0.08
3) Orientación diaria/Inspección de la pulsera/Instalación de la pulsera (n = 75)	0.09	0.03	0.06	0.30	0.09
4) Aplicar la escala Morse (n = 75)	0.13	0.03	0.07	0.22	0.12
5) Preparación del SAE (n = 75)	0.45	0.18	0.20	1.12	0.41
6) Aplicar el plazo de abandono (n = 75)	0.11	0.06	0.06	0.29	0.09
7) Preparar termo/pulso (n = 75)	0.12	0.07	0.06	0.29	0.09
8) Movilizar al paciente/asistir en la deambulación (n = 75)	0.11	0.01	0.10	0.12	0.11

*Tipo de conversión: R$ 5,46/US$ 1,00. Banco Central, con base en la tasa de cambio del 31/08/2024.

En el subproceso de asignación de TE, cada una de las tres actividades preventivas fue observada 96 veces en los tres turnos de TE. El tiempo medio empleado para realizar las tres actividades fue de 11,8 (DE = 3,80) minutos y la mediana fue de 11,10 minutos. Las actividades preventivas que llevaron más tiempo fueron: “1) Orientación diaria/Inspeccionar muñequera/Instalar muñequera/Barras elevadoras/Campanilla de posición” (4,5–DP = 1,9 minutos) y “3) Tomar notas de enfermería” (4,4–DP = 1,9 minutos); el tiempo medio empleado en realizar la actividad “2) Movilizar al paciente/Ayudar en la deambulación” fue de 2,0 (SD = 1,9) minutos. El coste total fue de 51,12 US$, con una MOD de 0,53 US$ (SD = 0,17) y una mediana de 0,50 US$. Según la Tabla 2, las actividades de con mayor CDM fueron “1) Orientación diaria/Inspeccionar pulsera/Instalar pulsera/Barras elevadoras/Campana de posición” (US$ 0,20 – DE = 0,09) y “3) Hacer notas de enfermería” (US$ 0,20 – DE = 0,07).

**Tabla 2:** Distribución de los costes, en dólares estadounidenses (US$*), de las tres actividades recomendadas para el mantenimiento del Protocolo de Prevención de Caídas en Adultos en la UICC realizadas por 26 TE – São Paulo, SP, Brasil, 2024.

Actividades (número de observaciones)	CDM	Desviación estándar	Coste mínimo	Máximo máximo	Mediana
1) Orientación diaria/Inspección de la pulsera/Instalación de la pulsera/Barras elevadoras/Campana de posición (n = 96)	0.20	0.09	0.09	0.54	0.18
2) Movilizar al paciente/asistir en la deambulación (n = 96)	0.13	0.08	0.03	0.41	0.12
3) Toma de notas de enfermería (n = 96)	0.20	0.07	0.05	0.38	0.21

*Tipo de conversión: R$5,46/US$1,00. Banco Central, basado en el tipo de cambio del 31/08/2024.

Considerando los conjuntos de actividades preventivas necesarias para el mantenimiento del “Protocolo de Prevenciónde Caídas de Adultos” en la UICC, realizados por la EnfA (CDM de US$ 1,24 – DE = 0,19) y TE (CDM de US$ 0,53 – DE = 0,17), el CDM total con MOD para los profesionales de enfermería fue de US$ 1,77 por paciente. Durante todas las observaciones de los conjuntos de actividades mencionados, el único material consumido fue la pulsera azul (costo unitario de US$ 0,01), totalizando US$ 1,71 correspondiente al consumo de 171 unidades.

Por último, cabe señalar que el costo directo mensual de materiales, establecido en la cuota de la UICC, para el mantenimiento del protocolo fue de US$ 10,10, de los cuales US$ 7,50 correspondieron a 750 unidades de pulseras azules y US$ 2,60 a cuatro unidades de marcadores de pizarra (costo unitario de US$ 0,65).

## DISCUSIÓN

Las actividades preventivas que forman parte de los subprocesos «funciones de la enfermera» y «funciones del técnico en enfermería», incluidas en el proceso de implementación y mantenimiento del «Protocolo de prevención de caídas en adultos» en la UICC, fueron realizadas por las enfermeras y los técnicos en enfermería, tal y como se había previsto; no se observaron aspectos relacionados con ellas que requirieran ajustes o reformulaciones.

En el área de la salud, mediante el mapeo de procesos, es posible identificar cuellos de botella, errores y oportunidades de mejora, lo que permite una toma de decisiones más informada y basada en datos. Por lo tanto, se trata de una herramienta esencial que contribuye a la mejora continua de la calidad, la seguridad y la eficiencia de los servicios ofrecidos^(6)^.

La creciente adopción de iniciativas de mejora continua de la calidad en la salud ha generado un aumento del interés en la investigación para profundizar su comprensión. Esto se debe a que tales iniciativas pueden tener un impacto significativo en la calidad de la asistencia sanitaria en varias áreas, con un enfoque en la mejora de la estructura, el proceso de prestación de servicios de salud, la mejora del bienestar del cliente y la reducción de la mortalidad^(12)^.

En esta perspectiva, la gestión basada en procesos es fundamental para garantizar una atención de calidad, optimizar los recursos y mejorar la experiencia, tanto de los pacientes como de los equipos de profesionales de la salud involucrados. Propone un enfoque para la mejora continua de los procesos con el fin de alcanzar los resultados deseados, contribuyendo al logro de los resultados asistenciales y administrativos en las organizaciones hospitalarias, con repercusiones favorables en los aspectos económico-financieros^(9)^.

Para la implementación del «Protocolo de Prevención de Caídas en Adultos» en la UICC, no se realizaron modificaciones estructurales, ya que la Unidad ya contaba con los recursos necesarios para la prevención adecuada de caídas. A través de este estudio, se demostraron inicialmente los costos asociados a la adquisición de materiales y a la inversión en la planificación y viabilidad de la capacitación de los profesionales de enfermería.

Es importante considerar que favorecer la capacitación, a través de la actuación imprescindible de la enfermera de prácticas asistenciales, encargada de impartir los programas específicos de formación, incrementó la prestación de cuidados al propiciar la mejora del desempeño de las actividades laborales, contribuyendo a la mejora continua de la calidad asistencial y la seguridad del paciente. La inversión en capacitación también ha permitido la asignación y el uso racional de los recursos, minimizando el desperdicio y dando como resultado la eficacia y efectividad de la aplicación de las actividades preventivas de caídas recomendadas, lo que se constató durante los momentos de observación no participante.

En relación con la MOD de EnfA y TE requerida para el mantenimiento del Protocolo, también se evidenció la inversión financiera del HGPM. Cabe destacar la pertinencia de la realización de actividades preventivas a pie de cama, ya sea en la gestión de la atención o en la prestación de cuidados directos al paciente. Se destaca la relevancia de la gestión de riesgos realizada diariamente por la EnfA. Antes de la implementación del Protocolo, el riesgo de caídas en la UICC se señalaba mediante esferas azules en la receta médica y en la identificación impresa junto a la cama del paciente, pero sin aplicar ninguna escala de evaluación del riesgo de caídas.

Tras la implementación, la gestión del riesgo se hizo más precisa e individualizada con la aplicación de la Escala de Caídas de Morse^(13)^. La aplicación adecuada de esta Escala es fundamental para la gestión del riesgo de caídas, ya que ayuda a identificar precozmente a las personas con mayor probabilidad de caerse, lo que permite al equipo de salud adoptar medidas preventivas de forma temprana al considerar el historial de caídas, el estado mental y la necesidad de ayuda, así como personalizar el enfoque individualizado, reduciendo el riesgo de incidentes y promoviendo un entorno más seguro^(14)^.

Cabe destacar que la individualización de la atención, a través de la SAE, orienta y fundamenta la actuación de los profesionales de enfermería, ya que la prescripción de cuidados exclusivos para la prevención de caídas en pacientes con riesgo medio y alto repercute favorablemente en un mejor uso del MOD por parte de los profesionales de enfermería.

La SAE es una herramienta valiosa que ayuda a definir el papel del enfermero, permitiéndole utilizar sus conocimientos técnicos, científicos y humanitarios en la prestación de los cuidados que requiere el individuo, y acreditar su ejercicio profesional con la puesta en práctica del Proceso de Enfermería, método que orienta el razonamiento clínico del enfermero para dirigir al equipo que gestiona en relación con los cuidados que se deben prestar al paciente, su familia y grupos específicos^(15)^.

Una revisión sistemática y un metaanálisis demostraron que las caídas, a pesar de considerarse un EA evitable, siguen siendo un problema común en los hospitales públicos y privados de todo el mundo. Se comprobó que, entre las intervenciones preventivas adoptadas (educación directa de pacientes y/o profesionales; modificaciones en el entorno de cuidados; dispositivos tecnológicos de asistencia; sistemas, modelos de servicio, contexto social, liderazgo, políticas o procedimientos para mitigar las caídas; rehabilitación, fisioterapia, actividades físicas u otros ejercicios terapéuticos; gestión de medicamentos; modificación de la dieta), la educación de los pacientes y de los profesionales de la salud consistió en la estrategia más favorable para evitar y reducir la ocurrencia de caídas hospitalarias^(16)^.

Así pues, se corrobora la importancia del trabajo en equipo y la práctica colaborativa para lograr buenos resultados en la prevención de caídas hospitalarias, pero para ello los profesionales de la salud deben apropiarse de las competencias necesarias para la acción colaborativa, destacando la comunicación con los pacientes/acompañantes y la comunicación interprofesional entre los miembros del equipo de salud de la unidad de hospitalización^(17)^.

Ante los elevados costos asociados a las caídas de personas mayores, en el periodo comprendido entre 2000 y 2020, un estudio realizado a partir del Sistema de Información Hospitalaria del Sistema Único de Salud brasileño destacó la necesidad de invertir en medidas más eficaces para prevenir y mitigar los daños derivados de las caídas en personas mayores, favoreciendo la prolongación de la vida con seguridad y calidad, reduciendo los riesgos de caídas aumentando las iniciativas de salud pública con prácticas inclusivas, como la adquisición de muebles o equipos de uso diario, lo que se traduce en una disminución del número de hospitalizaciones^(18)^.

En los Estados Unidos de América, un estudio de casos y controles con 900 635 pacientes investigó el costo de una caída en un entorno hospitalario y la relación costo-beneficio de la implementación de un programa de prevención de caídas (*Tailoring Interventions for Patient Safety* – Fall TIPS), analizando el periodo anterior, durante y posterior a su implementación. Se demostró que una caída sin lesiones generaba un costo de 62 521 dólares estadounidenses para la institución, de los cuales 35 365 dólares estadounidenses se clasificaron como costos directos; una caída con cualquier tipo de lesión correspondía a 64 526 dólares estadounidenses. El costo total de la implementación de Fall TIPS fue de 267,700.00 dólares y contribuyó a la prevención de 567 caídas, lo que supuso un ahorro total de hasta 22,036,714 dólares^(19)^.

Por último, además de la determinación de los costes relativos al proceso de implementación y mantenimiento del «Protocolo de Prevención de Caídas en Adultos», se corrobora que la prevención debe ser siempre una prioridad para los enfermeros^(20)^ y los técnicos de enfermería, independientemente de los costes asociados, con el fin de evitar los daños causados por la ocurrencia de este EA evitable, las consecuencias asistenciales derivadas de él y los costes intangibles para el paciente, que representan el cambio en la calidad de vida del individuo y las consecuencias de la propia enfermedad, como el dolor y el sufrimiento^(21)^.

### Implicaciones para la Práctica

Al poner de manifiesto los aspectos financieros asociados a la MOD de enfermeros, en puestos directivos y asistenciales, y de TE, así como a la adquisición de los recursos materiales necesarios para la implementación y el mantenimiento de un protocolo de caídas de adultos en la Unidad de Hospitalización, este estudio de microcosteo contribuye a la verticalización del conocimiento sobre el tema.

El método adoptado puede reproducirse en otros contextos hospitalarios y los resultados servirán de base para la toma de decisiones relativas a la asignación racional de los recursos humanos y materiales necesarios.

### Limitaciones del Estudio

Las observaciones de las actividades preventivas de caídas se realizaron únicamente por parte de enfermeras y técnicos en enfermería, en una sola Unidad de Internamiento, lo que constituye una limitación de este estudio. Para futuros estudios, se indica la relevancia de incluir a otros profesionales de la salud que también desarrollan acciones preventivas de caídas y ampliar el número de unidades.

## CONCLUSIÓN

El costo directo de la implementación del «Protocolo de prevención de caídas en adultos» en la UICC ascendió a 819,24 dólares estadounidenses, de los cuales US$218,44 correspondieron a la MOD de los profesionales de enfermería (enfermera de prácticas asistenciales, enfermera coordinadora, enfermera y técnico de enfermería) y US$600,80 correspondieron a la adquisición de materiales (rollos de pulseras azules, pizarras blancas y rotuladores para pizarras blancas).

Para el mantenimiento del Protocolo, se obtuvo un costo directo medio total de US$ 1,77/paciente en concepto de actividades de enfermería y US$ 10,10 en concepto de gastos mensuales en materiales. Las actividades preventivas realizadas por EnfA con mayor CDM fueron «5) Elaborar el SAE» (0,45 USD/DE = 0,18), «1) Anamnesis y examen físico» (0,13 USD/DE = 0,03) y «4) Aplicar la escala de Morse» (0,13 USD/DE = 0,03). Las actividades preventivas realizadas por los TE con mayor CDM fueron: «1) Orientación diaria/Inspeccionar pulsera/Colocar pulsera/Elevar barandillas/Colocar timbre» (0,20 dólares estadounidenses – DE = 0,09) y «3) Realizar anotaciones de enfermería» (0,20 dólares estadounidenses – DE = 0,07).

Se concluye que el conocimiento de los costes directos asociados a la MOD de los profesionales de enfermería y a la adquisición/reposición de recursos materiales puede servir de base para la toma de decisiones relativas a la asignación racional de los recursos necesarios para la viabilidad y el éxito del proceso de implementación y mantenimiento del «Protocolo de prevención de caídas en adultos».

## DISPONIBILIDAD DE DATOS

No aplicable. Se trata de un estudio de caso único, cuyos datos que respaldan los resultados no están disponibles públicamente debido a la presencia de información sensible y a la necesidad de preservar la confidencialidad de los participantes. Toda la información relevante para comprender los hallazgos se describe en el cuerpo del artículo.
